# Experimental Study on an Inclined Cylindrical Piezoelectric Energy Harvester

**DOI:** 10.3390/mi17030372

**Published:** 2026-03-19

**Authors:** Hao Li, Chongqiu Yang, Wenhui Li, Rujun Song, Xiaohui Yang

**Affiliations:** School of Mechanical Engineering, Shandong University of Technology, Zibo 255000, China

**Keywords:** vortex-induced vibration, piezoelectric energy harvester, inclined cylinder, wider operating frequency band

## Abstract

Energy harvesting plays a pivotal role in enabling sustainable power supply for the Internet of Things and distributed sensor networks, particularly for low-power devices. Piezoelectric energy harvesters based on vortex-induced vibrations offer a promising solution for low-wind-speed applications, yet their performance is constrained by limited bandwidth and sensitivity to wind speed variations. This study addresses these limitations by proposing a novel multi-parameter adjustable piezoelectric energy harvester featuring an inclined cylindrical bluff body. By systematically tuning the inclination angle and installation position, the device achieves substantial performance improvements. Experimental results indicate that the optimized configuration yields a wider operational frequency band and enhanced energy conversion efficiency. Through the experimental results, we discovered the existence of the double-peak phenomenon and the plateau phenomenon. The voltage value of the second peak can reach up to 122.4% of the maximum voltage of the first peak. The duration of the maximum plateau phase can maintain between the wind speed of 2.3 m/s and 5.7 m/s.

## 1. Introduction

Driven by the global energy transition and carbon neutrality targets, the importance of renewable energy development and utilization has become increasingly prominent. As one of the most promising clean energy sources, the traditional utilization of wind energy mainly relies on horizontal or vertical axis wind turbines. However, the efficiency bottleneck of such devices in low-wind-speed environments, as well as problems such as noise pollution and maintenance costs, have prompted researchers to explore new wind energy capture technologies. Wind vibration power generation provides new ideas to solve these problems by combining the aeroelastic effect with energy conversion mechanisms (e.g., piezoelectric, electromagnetism, etc.).

In the current renewable energy technology system, solar energy [1,2], wind energy [3,4], geothermal energy [5,6] and tidal energy [7,8] have their own characteristics. Among them, wind-induced vibration energy capture technologies based on aerodynamic instability can be categorized into vortex-induced vibration (VIV) [9,10], galloping [11,12], and flutter [13,14], based on the different mechanisms of action. These technologies realize the conversion of energy forms from vibration energy to electrical energy through physical mechanisms such as piezoelectric conversion [15,16], electromagnetic induction [17,18] or electrostatic conversion [19,20,21]. It is worth noting that the wind-induced vibration power generation technology not only inherits the clean characteristics of traditional wind energy utilization, but also breaks through the application limitations of traditional technology through structural innovation, which provides a new technological path for constructing distributed micro-energy networks.

In order to broaden the effective wind speed bandwidth or to increase the efficiency of the energy harvester, a lot of theoretical, computational and experimental studies have been carried out and various improvements have been proposed. Common approaches are to change the aerodynamic cross-sectional shape of the blunt body and add removable attachments. Wang et al. [21] propose three types of hybrid cross-section blunt bodies by combined vortex-induced vibration (VIV) and galloping, integrate the characteristics of circular (VIV advantage) and square (galloping advantage) bodies, which significantly improves the efficiency of a wind energy harvester and solves the problem of poor wind speed adaptability of traditional methods. Zhou et al. [22] propose a piezoelectric energy harvester with a bionic swallowtail V-shaped additional structure. By optimizing the blunt body design, the starting wind speed is significantly reduced, the voltage output is increased by 167%, and the power is increased by 7.16 times, which provides an efficient solution for wind energy harvesters in low-wind-speed environments. Zhang et al. [23] introduce two repulsion magnets at the bottom of the cylinder and on the supporting structure. By adjusting their relative positions, the natural frequency and damping ratio of the system can be adjusted, thereby broadening the synchronization area and increasing the output power. Man et al. [24] present a three-stable piezoelectric energy harvester driven by a dynamic amplifier. By simultaneously amplifying the rotation and lateral displacement of the cantilever beam, it significantly enhances the inter-well motion response. It achieves wider frequency band and lower excitation threshold for energy collection under low-frequency and small-amplitude vibrations, providing an efficient solution for powering low-power devices such as those in the Internet of Things.

In addition, the researchers took a different approach and systematically studied the inclined cylindrical blunt bodies in a wind energy harvester by innovative adjusting of the installation angle of the structure. For instance, Javed and Abdelkefi’s [25] research indicates that vertical (0°) or slightly positively inclined (≤+15°) square columns are the optimal configuration for a vibration energy harvester, as they can achieve the maximum output power and the lowest startup wind speed. Reverse inclination and positive inclination greater than +15° will significantly reduce the system response and are not suitable for efficient energy harvesters. Shirakasi et al. [26] found that an increase in the inclination angle of the cylinder would significantly affect its wake characteristics: on the one hand, it would lead to an irregular vortex shedding process; on the other hand, it would cause a continuous decrease in the vortex shedding frequency. Subsequently, Matsumoto [27] reviewed various vortex shedding phenomena generated by blunt bodies in fluids and their influence on structural vibrations, and discussed the classification of vortices, vibration mechanisms and control methods. Franzini et al. [28] indicate that, as the inclination angle of the cylinder increases, the vortex shed mechanism will exhibit more irregular characteristics. Wang et al. [29] found that the inclination can significantly broaden the effective wind speed band of vortex-induced vibration. When α = 60°, the bandwidth increases by more than 229%. When α changes from 25° to 40°, it will trigger torsional vibration, which couples with lateral bending to form a “bimodal” output. Hu et al. [30] analyzed the effect of different tilt angles on the vibration response by wind tunnel experiments and pressure measurements. It was found that an increase in the forward inclination angle significantly reduces the response; a small backward inclination angle of −5° or −10° actually increases the response. When the angle is ≥−15° for a large backward inclination, it suppresses the vibration.

In summary, previous research on inclined cylinders focused solely on varying the inclination angle, with the cylinder always fixed at the middle position. In contrast, our study introduces the installation position (P) as an additional degree of freedom, i.e., the point where the cylinder is attached to the cantilever beam. This parameter significantly alters the dynamic coupling between bending and torsional modes, and leads to distinct phenomena such as double peaks and plateau regions, which have not been reported in angle-only studies. In this study, four different inclination angles α (20°, 30°, 40°, 50°), and five different positions (P1–P5) were experimentally tested in an experimental wind tunnel for performance evaluation. The inclined cylinder has an impact on the vibration motion state of the energy harvester.

## 2. Experimental Setup and Basic Theory

**Figure 1:**
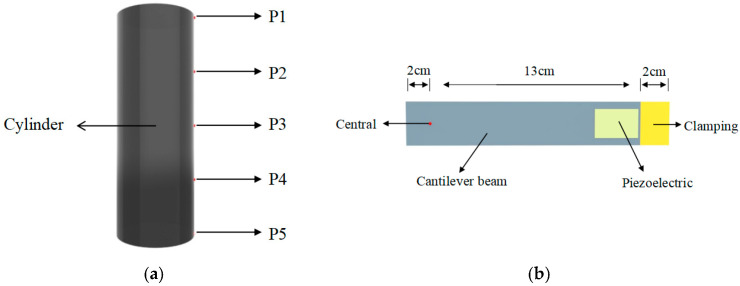
Schematic diagram of the physical model of the PEH. (**a**) 5 positions on the cylinder; (**b**) piezoelectric beam component.

The proposed PEH was constructed with a cylinder and a cantilever beam, as shown in Figure 1.

Figure 1a shows the 5 positions on the cylinder: the midpoint of the lateral side of the cylinder was named as P3 and the upper end as P1, we took the midpoint between P1 and P3 and named it P2, P4 and P5 were taken and named in the same way, by which the cylinder was divided into five positions. The cylinders had a diameter of 4.7 cm and the length of the cylinders was 13cm.

Figure 1b shows the piezoelectric beam, the red dot indicates the midpoint of the piezoelectric beam in the width, and the five position points P1–P5 of the cylinder were always kept to coincide with the midpoint of the piezoelectric beam in the experimental process. The length of the beam from the red dot to the front end, which was the part embedded in the cylinder, was 2 cm; the length from the red dot to the clamping end was 13 cm; and the rightmost end was the clamping part of 2 cm.

**Figure 2:**
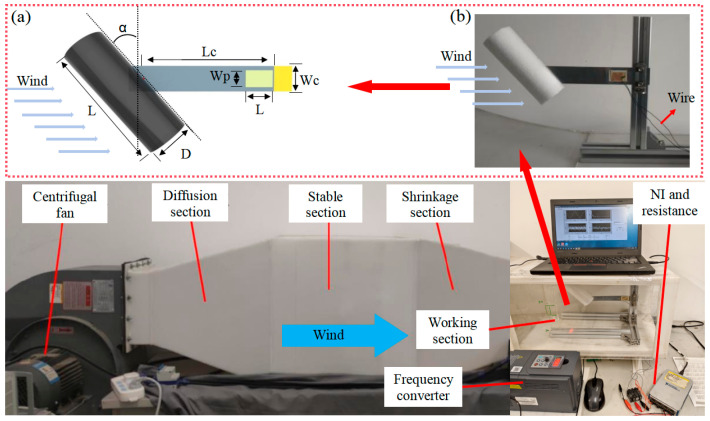
Schematic diagram of wind tunnel test platform. (**a**) schematic diagram of physical parameters of the harvester; (**b**) experiment prototype.

Figure 2 shows the schematic diagram of the wind tunnel test platform. The wind speed could be regulated from 1 m/s to 6 m/s precisely. Its main components include: a centrifugal fan, a diffusion section, a stable section, a shrinkage section, a frequency converter, and a working section. The length, width and height of the wind tunnel test platform were 2120 mm, 500 mm and 500 mm respectively. The three-dimensional dimensions of the experimental section were 500 mm × 300 mm × 280 mm. This volume was sufficient to place various small PEH prototypes for experimental tests.

It is worth noting that while PZT was selected for this study due to its high electromechanical coupling coefficient, which ensures clear validation of the structural design, its lead content raises concerns regarding environmental sustainability and biocompatibility [31]. Recent advances in green piezoelectric materials offer promising alternatives. For instance, piezoelectric biomaterials and hybrid devices have demonstrated intrinsic biocompatibility, biodegradability, and a reduced environmental footprint [32,33,34]. Although the current work focuses on structural optimization using PZT, future iterations of the inclined cylinder harvester could benefit from integrating such sustainable materials to better align with the requirements of implantable and eco-friendly electronics. Considering both the price and the preparation process, PZT-5H (Produced by Baoding Hongsheng Electronic Equipment, Baoding, China) was adopted. In the experiment, the PZT was polarized in d31 mode. To prepare the prototype, use sandpaper to polish the oxide layer on the surface of the beam. Then, use glue to attach the piezoelectric sheet to the beam. Finally, use a pressure clamp to hold it for 24 h to squeeze out the excess glue. The adhesive used between the piezoelectric sheet and the beam was 1309 ergo glue (made in Switzerland), as it is strong and conductive. Most of the glue was squeezed out, and only a small amount of glue played a fixing role. Therefore, during the data analysis stage of the experiment, we disregarded the influence of the glue.

The physical schematic diagram of the proposed PEH is shown in Figure 2a, and the specific parameters are listed in Table 1. Figure 2b is the experimental PEH; the cantilever beam was made of thin aluminum sheet while the cylinder was made of foam material. The cylinder and the piezoelectric beam were closely clamped at the 5 positions with a certain angle α of 20°, 30°, 40° and 50°. This study mainly focuses on a cylindrical object with a diameter of 4.7 cm. Table 1 shows the values of structural and material parameters of the physical model.

The piezoelectric element was electrically connected to an NI data acquisition card, which transmits the acquired voltage signals to a computer for real-time monitoring and storage, as shown in Figure 2. Wind speed regulation is achieved through a variable-frequency driver, enabling precise control over the flow conditions. This setup ensures accurate measurement of the electromechanical response under varying aerodynamic excitation. In addition, in the experiments, a fixed load resistance of 220 kΩ was adopted to ensure comparability across all test conditions to focus on the voltage output characteristics due to the effects of the inclination angle and installation position on the harvester’s performance under varying wind speeds.

The piezoelectric beam employed in the energy harvester comprises an elastic substrate layer and a piezoelectric patch. The piezoelectric patch is affixed at the clamped end of the elastic substrate, adjacent to the fixed boundary. To facilitate mathematical modeling and dynamic analysis of the inclined piezoelectric energy harvester, the following assumptions are established:

(1) The support layer and the piezoelectric layer are closely bonded, and the influence of the adhesive between them is not considered.
(2) The elastic piezoelectric beam cannot be elongated during the deformation process.
(3) The piezoelectric quantity fully conforms to the Euler–Bernoulli beam principle.
(4) The direction of the electric field intensity of the piezoelectric sheet is perpendicular to the bonding surface of the piezoelectric beam and is uniformly distributed along the vertical plane.
(5) The incoming flow direction is regarded as a uniform and stable fluid field everywhere.
(6) The cylinder connected to the free end is regarded as a rigid body, and the deformation of the cylinder is not considered.

For clear and concise expression in the modeling process, the following regulations are made in this paper:

(1) “′” represents the partial derivative of the function with respect to the arc length x;
(2) “·” represents the partial derivative of the function with respect to time;
(3) Subscript “s” represents the support layer, and subscript “p” represents the piezoelectric layer.

When the inclined piezoelectric energy harvester is placed in the flow field, its dynamic process is actually a complex system involving the coupling of multiple physical fields. Firstly, under the action of wind-induced vibration, the swing of the cylinder will cause the piezoelectric beam to deform, and then through the positive piezoelectric effect, it will affect the piezoelectric material to generate voltage. However, these voltages will feedback to the piezoelectric beam itself through the inverse piezoelectric effect, thereby affecting the vibration form and amplitude of the beam. Therefore, when modeling the entire system, the electromechanical coupling effect must be considered first. There is a continuous interaction between the cylinder and the surrounding flow field. The flow field force drives the vibration of the cylinder, while the movement of the cylinder will in turn change the structure of the flow field. In summary, the modeling of the inclined piezoelectric energy harvester must fully consider the coupling relationship and interaction among the flow field, solid structure and electric field. The analysis of the multi-field coupling system of the flow field, solid structure and electric field is the key basis for accurately predicting and optimizing its performance. The constitutive equation of the equivalent fluid–solid–electric coupling of the inclined piezoelectric interactive device is:

(1) $$\left\{\begin{array}{l} M\ddot{\omega }(x_{o},t)+C\dot{\omega }(x_{o},t)+K\omega (x_{o},t)+\Theta V(t)=F(t)\\ \frac{V(t)}{R_{L}}+C_{p}\dot{V}(t)-\Theta \dot{\omega }(x_{o},t)=0 \end{array}\right.$$

M—Equivalent mass (Kg)
C—Equivalent damping
K—Equivalent stiffness (N/m)
Θ—Mechanical–electrical coupling coefficient (N/V)
$R_{L}$—External resistance (Ω)
$C_{p}$—Equivalent capacitance of piezoelectric sheet (F)
$\omega (x_{o},t)$—Transverse displacement of equivalent point O at time t (m)
V(t)—Voltage across the external resistance (V)
F(t)—Equivalent vortex-induced lift (N)

**Figure 3:**
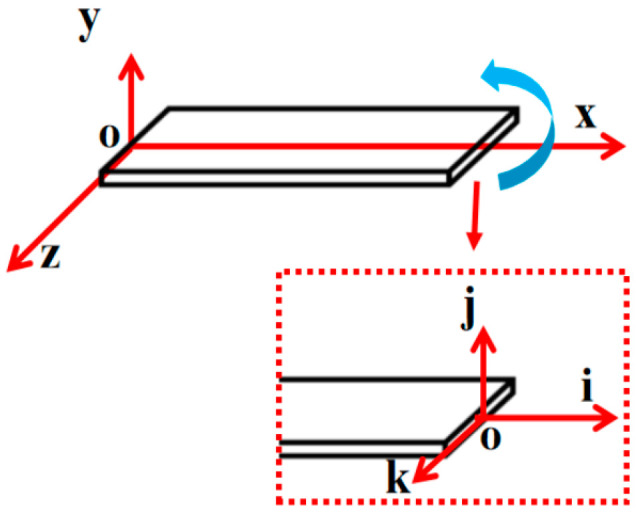
Coordinate system diagram.

Since the piezoelectric beam undergoes both bending and torsion simultaneously, two coordinate systems are required to describe the vibration displacement and torsional displacement of the piezoelectric beam, as shown in Figure 3; O−xyz is the fixed coordinate, fixed on the clamping side of the piezoelectric beam, serving as the reference coordinate system for describing the bending motion; O−ijk is the reference coordinate system for describing the torsional motion at the free end of the piezoelectric beam, and the large arrow indicates the torsional direction of the piezoelectric beam.

**Figure 4:**
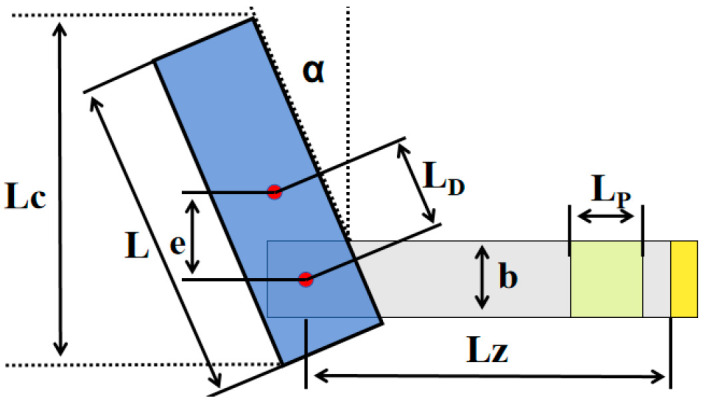
The front view of the inclined piezoelectric energy harvester device.

As shown in Figure 4, this is the front view of the inclined piezoelectric energy harvester device. The data of each part in the figure is consistent with that in the previous table. Here, e represents the vertical distance from the center of the cylinder to the center of the piezoelectric sheet, $L_{D}$ represents the straight-line distance from the center of the cylinder to the center of the beam, $L_{C}$ represents the actual length of the wind column that receives the incoming flow impact, and the equivalent beam length is represented by $L_{s}=L_{z}+L_{D}sin\alpha$.

**Figure 5:**
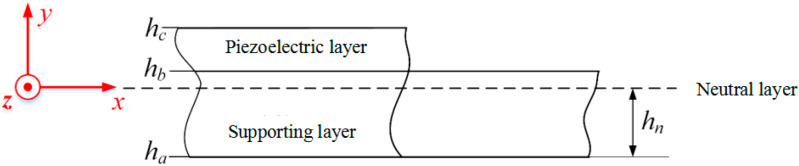
The schematic diagram of the neutral axis.

Due to the different thicknesses of the supporting layer and the piezoelectric layer, when the piezoelectric charge undergoes deformation, it will cause the neutral layer to not be at the geometric center position. Therefore, it is necessary to determine the neutral layer of the piezoelectric charge and the schematic diagram of the neutral axis, as shown in Figure 5.

The position of the neutral axis in the piezoelectric beam can be determined according to the principle of stiffness equivalence. The specific representation is as follows:

(2) $$h_{n}=\frac{(h_{p}+h_{s})E_{p}h_{p}}{2(E_{p}h_{p}+E_{s}h_{s})}+\frac{h_{s}}{2}$$

From the above figure, the expressions for $h_{a}$, $h_{b}$, and $h_{c}$ are respectively

(3) $$h_{a}=-h_{n}$$

(4) $$h_{b}=h_{s}-h_{n}$$

(5) $$h_{c}=h_{s}+h_{p}-h_{n}$$

In order to establish the control equations for the energy harvester system, we first calculate the total kinetic energy, total potential energy, and virtual work. The total kinetic energy of the energy harvester system T consists of the kinetic energy $T_{b}$ of the cantilever beam, the kinetic energy of the attached cylinder, and the additional fluid kinetic energy T_c_ caused by the added fluid mass.

(6) $$T_{b}=\frac{1}{2}\rho_{s}\int_{V_{s}}\left[{\dot{w}}^{2}(x,t)+I_{si}{\dot{\varphi }}^{2}(x,t)\right]dV_{s}+\frac{1}{2}\rho_{p}\int_{V_{p}}\left[{\dot{w}}^{2}(x,t)+I_{pi}{\dot{\varphi }}^{2}(x,t)\right]dV_{p}$$

The total kinetic energy $T_{c}$ includes translational kinetic energy and rotational kinetic energy, and its expression is

(7) $$T_{c}=\frac{1}{2}[M_{c}+\pi (\frac{D}{2}{)}^{2}L{c\rho }_{f}]{\left[\dot{w}(L_{s},t)+\frac{D}{2}{\dot{w}}^{\prime }(L_{s},t)\right]}^{2}+\frac{1}{2}(\left[M_{c}\left(\frac{L_{c}^{2}}{12}+e^{2}\right)+M_{f}\left(\frac{L_{c}^{2}}{12}+e^{2}\right)\right]){\dot{\varphi }}^{2}(L_{s},t)$$

The total potential energy U of the energy collection system consists of the bending–torsion potential energy Us of the piezoelectric beam, the electric potential energy Ue, and the gravitational potential energy Ug.

(8) $$Ui=\frac{1}{2}\int_{V_{s}}\left(\sigma^{s}\varepsilon^{s}+\tau^{s}\gamma^{s}\right)dV_{s}+\frac{1}{2}\int_{V_{p}}\left(\sigma^{p}\varepsilon^{p}+\tau^{p}\gamma^{p}\right)dV_{p}$$

When the energy collector vibrates, the gravitational potential energy Ug generated by the center of gravity of the cylinder’s movement can be expressed by the following formula:

(9) $$Ug=\frac{M_{c}ge\varphi^{2}(L_{s},t)L_{c}}{2}$$

The total virtual work includes the virtual work of the load resistance $\delta W_{R}$, the lift $\delta W_{l}$, the fluid resistance $\delta W_{cf}$, and the mechanical damping $\delta W_{cm}$. These parameters are given by the following formula.

(10) $$\delta W_{R}=-V(t)\delta Q(t)$$

(11) $$\delta W_{cm}=-\int_{0}^{L_{s}}\left[c_{mb}\dot{w}(x,t)\delta w(x,t)+c_{mt}\dot{\varphi }(x,t)\delta \varphi (x,t)\right]dx$$

(12) $$\delta W_{cf}=-c_{f}\dot{\varphi }(L_{s},t)e\delta \varphi (L_{s},t)-c_{f}L_{c}\left[\dot{w}(L_{s},t)+\frac{D}{2}{\dot{w}}^{\prime }(L_{s},t)\right]\delta \left[w(L_{s},t)+\frac{D}{2}\dot{w}(L_{s},t)\right]$$

(13) $$\delta W_{l}=F(t)\left\{\delta \left[w(L_{s},t)+\frac{D}{2}w^{\prime }(L_{s},t)\right]+e\delta \varphi (L_{s},t)\right\}$$

(14) $$c_{f}=\frac{C_{D}\rho_{f}DU}{2}$$

F(t) represents the aerodynamic lift caused by eddy currents, which is given by the following formula:

(15) $$F(t)=\frac{C_{L}(t)\rho_{f}L_{c}DU^{2}}{2}$$

To describe the wake situation near the cylindrical body, the expression $q(t)=2C_{L}(t)/C_{L0}$, is introduced, and it is calculated using the van der Pol equation.

(16) $$\ddot{q}+\varepsilon \omega_{f}(q^{2}-1)\dot{q}+\omega_{f}^{2}q=\left(\frac{A}{D}\right)\left[\ddot{w}(L_{s},t)+\frac{D}{2}\ddot{w^{\prime }}(L_{s},t)\right]$$

Here, ωf is expressed as ωf=2πSt/D.

Next, in order to study the dynamic response of the piezoelectric energy harvester system with bending–torsional deformation, we employed the Galerkin method to establish a reduced-order model, which is presented in the following form:

(17) $$\begin{array}{rr} w(x,t) & =\sum_{i=1}^{n}\phi_{wi}(x)r_{i}(t)\\ \varphi (x,t) & =\sum_{i=1}^{n}\phi_{\varphi i}(x)r_{i}(t) \end{array}$$

Since the piezoelectric layer does not completely cover the base layer, the bending mode function and the torsion mode function are divided into two parts. The shape expression $\phi_{wi}(x)$ of the bending model can be determined as

(18) $$\phi_{wi}=\left\{\begin{array}{ll} \phi_{wi1}(x)=A_{1}\sin\lambda_{wi1}x+B_{1}\cos\lambda_{wi1}x & \\ \;\;\;\;\;\;\;\;\;\;\;\;\;+C_{1}\sinh\lambda_{wi1}x+D_{1}\cosh\lambda_{wi1}x, & 0\leq x<L_{s}-L_{p}\\ \phi_{wi2}(x)=A_{2}\sin\lambda_{wi2}x+B_{2}\cos\lambda_{wi2}x & \\ \;\;\;\;\;\;\;\;\;\;\;\;\;+C_{2}\sinh\lambda_{wi2}x+D_{2}\cosh\lambda_{wi2}x, & L_{s}-L_{p}\leq x\leq L_{s} \end{array}\right.$$

Similarly, the shape expression $\phi_{\varphi i}(x)$ of the transformation model can be determined as

(19) $$\phi_{\varphi i}=\left\{\begin{array}{cc} \phi_{\varphi i1}(x)=A_{3}\sin\lambda_{\varphi i1}x+B_{3}\cos\lambda_{\varphi i1}x, & 0\leq x<L_{s}-L_{p}\\ \phi_{\varphi i2}(x)=A_{4}\sin\lambda_{\varphi i2}x+B_{4}\cos\lambda_{\varphi i2}x, & L_{s}-L_{p}\leq x\leq L_{s} \end{array}\right.$$

By introducing the Lagrange equation, the coupled control equations of the fluid–solid–electric energy harvester are obtained.

(20) $$\left\{\begin{array}{l} \frac{\partial }{\partial t}\left(\frac{\partial L}{\partial \dot{r}}\right)-\frac{\partial L}{\partial r}=\frac{\delta W}{\delta r}\\ \frac{\partial }{\partial t}\left(\frac{\partial L}{\partial \dot{\gamma }}\right)-\frac{\partial L}{\partial \gamma }=\frac{\delta W}{\delta \gamma } \end{array}\right.\;\dot{\gamma }=V$$

Based on the orthogonality conditions of different modal functions, the mass and stiffness matrices were derived.

(21) $$\begin{array}{c} \begin{array}{ll} \rho_{s} & \int_{0}^{L_{p}}\int_{-\frac{b_{s}}{2}}^{\frac{b_{s}}{2}}\int_{h_{a}}^{h_{b}}\left[\phi_{w1}^{2}(x)+(y^{2}+z^{2})\phi_{\varphi_{1}}^{2}(x)\right]dydzdx\\ & +\rho_{p}\int_{0}^{L_{p}}\int_{-\frac{b_{p}}{2}}^{\frac{b_{p}}{2}}\int_{h_{b}}^{h_{c}}\left[\phi_{w1}^{2}(x)+(y^{2}+z^{2})\phi_{\varphi_{1}}^{2}(x)\right]dydzdx\\ & +\rho_{s}\int_{L_{p}}^{L_{s}}\int_{-\frac{b_{s}}{2}}^{\frac{b_{s}}{2}}\int_{-\frac{h_{s}}{2}}^{\frac{h_{s}}{2}}\left[\phi_{w2}^{2}(x)+(y^{2}+z^{2})\phi_{\varphi_{2}}^{2}(x)\right]dydzdx\\ & +M{\left[\phi_{w2}(L_{s})+\frac{D}{2}{\phi^{\prime }}_{w2}(L_{s})\right]}^{2}+J\phi_{\varphi_{2}}^{2}(L_{s})=1 \end{array} \end{array}$$

(22) $$\begin{array}{ll} & \int_{0}^{L_{p}}\int_{-\frac{b_{s}}{2}}^{\frac{b_{s}}{2}}\int_{h_{a}}^{h_{b}}\left[E_{s}y^{2}{\phi_{w1}}^{\prime \prime 2}(x)+G_{s}\left(y^{2}+z^{2}\right){\phi_{\varphi 1}}^{\prime 2}(x)\right]dydzdx\\ & +\int_{0}^{L_{p}}\int_{-\frac{b_{p}}{2}}^{\frac{b_{p}}{2}}\int_{h_{b}}^{h_{c}}\left[E_{p}y^{2}{\phi_{w1}}^{\prime \prime 2}(x)+G_{p}\left(y^{2}+z^{2}\right){\phi_{\varphi 1}}^{\prime 2}(x)\right]dydzdx\\ & +\int_{L_{p}}^{L_{s}}\int_{-\frac{b_{s}}{2}}^{\frac{b_{s}}{2}}\int_{-\frac{h_{s}}{2}}^{\frac{h_{s}}{2}}\left[E_{s}y^{2}{\phi_{w2}}^{\prime \prime 2}(x)+G_{s}\left(y^{2}+z^{2}\right){\phi_{\varphi 2}}^{\prime 2}(x)\right]dydzdx=\omega^{2} \end{array}$$

Here, L represents the Lagrange quantity. Subsequently, the reduced-order state equation is obtained as follows:

(23) $$\begin{array}{rr} \ddot{r}(t)+2\omega l\dot{r}(t)+\left(ð+\omega^{2}\right)r(t)-\lambda {\left[r^{2}(t)+\gamma^{2}\right]}^{-5/2}r(t)+Br^{3}(t)-\theta V(t) & =F \end{array}$$

(24) $$\frac{V(t)}{R}+C_{p}\dot{V}(t)+\theta \dot{r}(t)=0$$

Among them, the system damping ratio l can be determined by the free vibration attenuation method to simplify the calculation process. By introducing state variables, the above nonlinear differential equation can be solved, and it can be expressed by the following formula:

(25) $$X=\left[\begin{array}{c} X_{1}\\ X_{2}\\ X_{3} \end{array}\right]=\left[\begin{array}{c} r(t)\\ \dot{r}(t)\\ V(t) \end{array}\right]$$

Based on the previous formula, it can be concluded that

(26) $$\dot{X}=\left[\begin{array}{c} {\dot{X}}_{1}\\ {\dot{X}}_{2}\\ {\dot{X}}_{3} \end{array}\right]=\left[\begin{array}{c} X_{2}\\ -2\omega lX_{2}-(ð+\omega^{2})X_{1}+\lambda (X_{1}^{2}+\gamma^{2}{)}^{-5/2}X_{1}-BX_{1}^{3}+\theta X_{3}+F\\ -1/(C_{p}R)X_{3}-\theta /C_{p}X_{2} \end{array}\right]$$

## 3. Results and Discussion

In this study, a controlled variable approach was adopted. For each experimental configuration, the inclination angle was held constant while the installation position was systematically varied. The following key performance indicators are introduced to characterize the harvester’s behavior: the effective wind speed range, the double-peak phenomenon, and the plateau region.

1. The effective wind speed range is defined as the interval between the cut-in wind speed, at which the root mean square (RMS) voltage first exceeds 1 V, and the cut-out wind speed, at which the voltage drops below 1 V.
2. The double-peak phenomenon refers to a response pattern where, after the voltage reaches its first maximum, it drops significantly—approaching near-zero levels—before rising again to a second peak as the wind speed continues to increase.
3. The plateau region is defined as a wind speed interval of at least 1 m/s over which the output voltage fluctuates by less than 1 V. This region is characterized by a stable voltage output despite variations in wind speed, making the harvester particularly suitable for applications subject to fluctuating wind conditions.

**Figure 6:**
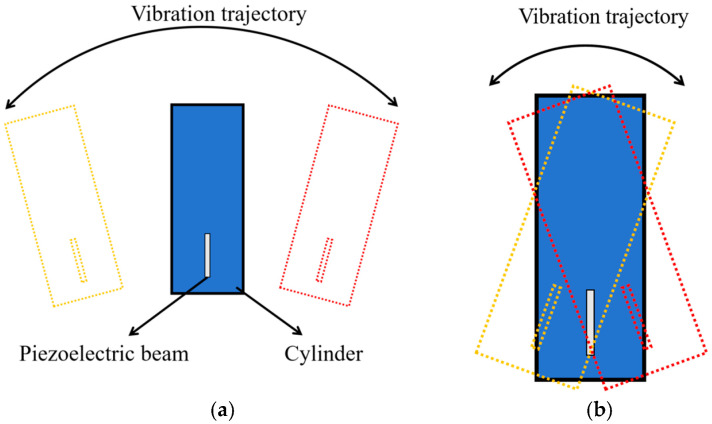
The movement posture of the cylinder. (**a**) Low-wind-speed movement posture; (**b**) High-speed movement posture.

As illustrated in Figure 6a, the yellow and red contours represent the extreme positions of the cylinder during its left and right oscillations. At low wind speeds, the cylinder predominantly vibrates in the lateral bending mode, which is characterized by translational motion—i.e., the upper and lower ends of the cylinder move in parallel and in the same direction. Due to the eccentric installation of the piezoelectric beam, its deformation exhibits an arc-shaped trajectory. In this configuration, the piezoelectric sheet undergoes primarily bending deformation, accompanied by a minor torsional component. The generation of voltage mainly comes from the lateral bending of the piezoelectric beam. As shown in Figure 6b, as the wind speed gradually increases, the cylindrical body will undergo torsional vibration, presenting a combined state of bending and torsion. This phenomenon becomes more pronounced in slender cylindrical bodies. In the superimposed vibration mode of the cylindrical body, the upper and lower ends of the blunt body move in opposite directions, causing the beam attached to the blunt body to undergo torsional deformation, while the entire blunt body will undergo translational motion, causing the piezoelectric beam to deform and bend. The torsion of the blunt body will weaken the bending deformation of the piezoelectric beam. Due to the eccentricity in the installation of the piezoelectric beam, during the vibration process, the torsional center of the cylindrical body will move. When the installation position is at the center of the cylinder, the torsional center is at the middle position of the cylinder. When the installation position is connected to the lower end of the cylindrical body, the torsional center will move upward, enabling the piezoelectric beam to obtain more deformation in the horizontal direction. Compared to the installation state shown in Figure 6a, the lateral bending vibration of the harvester will slightly decrease, while the torsional vibration will significantly increase. Power generation still mainly relies on the lateral movement of the piezoelectric beam. In general, the energy generated by torsional vibration is lower than that produced by bending vibration. However, due to the eccentric installation of the piezoelectric vibrating beam, the bending vibration is enhanced to a certain extent, leading to an increase in voltage output.

**Figure 7:**
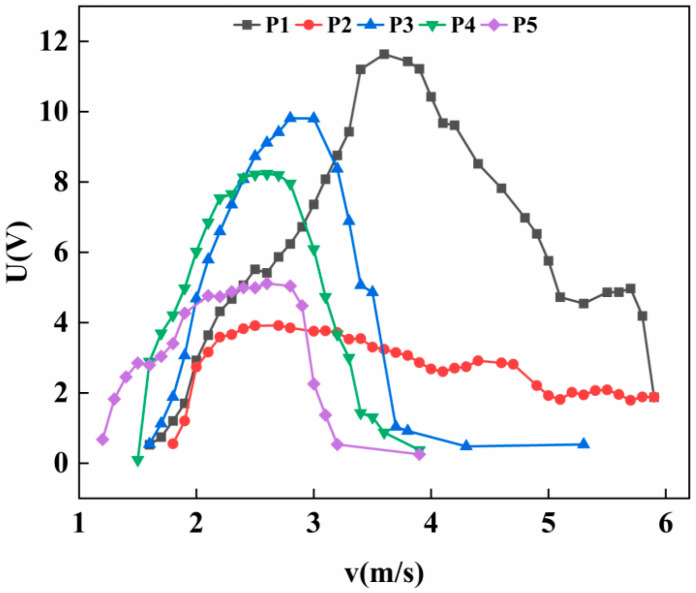
Variation of voltage with wind speed at 20° with different positions.

As shown in Figure 7, the output voltage of the inclined cylinder at position P1 initially increases with wind speed, reaching a peak before declining. After a slight recovery at approximately 5.3 m/s, the voltage subsequently drops again. In contrast, the cylinder at position P2 exhibits a pronounced plateau region. The voltage reaches a maximum of 3.91 V at a wind speed of 2.5 m/s. As the wind speed increases further, the voltage fluctuates and gradually decreases at an extremely slow rate. By the end of the test, the voltage measures 1.88 V, representing a reduction of only 2.03 V (approximately 51%) over a wind speed increase of 3.5 m/s. In comparison, the output voltages at other positions decline sharply to near zero over the same range. The plateau phase at position P2 is sustained for a wind speed interval exceeding 3 m/s. For positions P3 and P4, the voltage responses exhibit typical vortex-induced vibration characteristics, with the output first rising and then falling as wind speed increases. At position P5, the voltage shows a brief drop during the initial rise, followed by a relatively short plateau period, after which it rapidly declines to zero.

Under the experimental wind speed range of 0–6 m/s, the inclined cylinder at position P1 exhibited the highest output voltage and the widest effective wind speed range. For position P2, the voltage did not drop to zero even at the maximum tested wind speed, suggesting that its operational range may extend further if the wind speed increases beyond the current limit. Judging from the slow decline trend of the voltage, the inclined cylinder at position P2 has great potential.

Regarding the causes of the plateau phenomenon, when the wind speed reaches a certain threshold, the vortex shedding frequency approaches the natural frequency of the system, causing the system to enter a locked state and start vibrating. Due to the nonlinear damping of the system increasing rapidly with amplitude, or due to the modal competition dispersing the energy, the vibration amplitude cannot continue to increase and is limited to a relatively low level. As the wind speed continues to increase, but the damping balance keeps the strain and output voltage of the piezoelectric beam basically unchanged, a plateau phenomenon is formed. Due to the incomplete symmetry of the tilt of the wind column and the eccentricity of the connection point, the bending vibration may transfer part of the energy to the torsional mode or other higher-order modes through geometric nonlinear coupling. These leaked energies no longer contribute to the bending vibration, preventing the amplitude of the bending vibration from continuing to increase, and thus manifesting as a plateau period in the macroscopic voltage.

**Figure 8:**
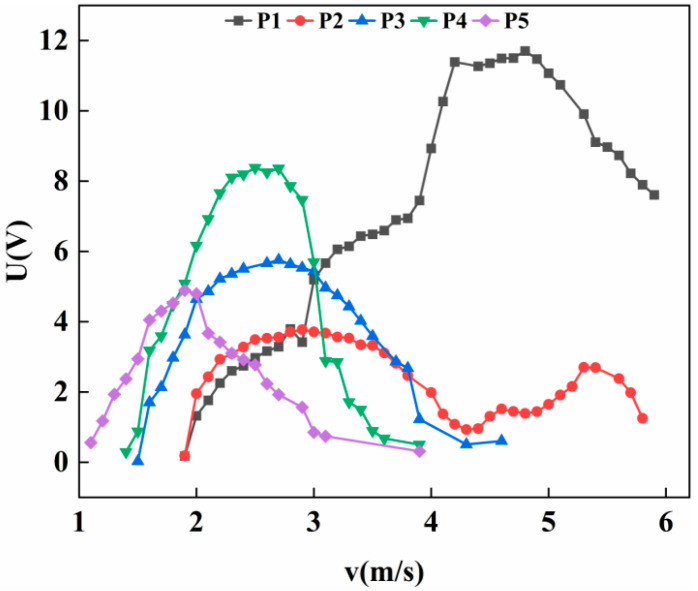
Variation of voltage with wind speed at 30° with different positions.

As shown in Figure 8, for the cylinder fixed at an inclination angle of 30°, the output voltage at position P1 increases monotonically with wind speed, followed by a brief period of stability. However, as the wind speed increment during this phase does not exceed 1 m/s, it does not qualify as a plateau region. Subsequently, the voltage begins to decline. By the end of the test, the voltage at position P1 remains relatively high, although the data curve exhibits a clear and consistent downward trend. At position P2, the voltage initially rises and then drops with increasing wind speed. As the wind speed continues to increase, a secondary rise in voltage is observed, resulting in a double-peak phenomenon. For positions P3, P4, and P5, the output responses are characteristic of vortex-induced vibration, with the voltage first increasing to a peak and then decreasing as the wind speed progresses.

Under the experimental wind speed range of 0–6 m/s, the inclined cylinder at position P1 yielded the highest output voltage and maintained a relatively high voltage level by the end of the test. This indicates that its effective wind speed range could be further extended if the wind speed continues to increase. For the cylinder at position P2, the first peak reaches a maximum voltage of 3.78 V, while the second peak attains 2.7 V—approximately 71.4% of the first peak’s magnitude. The presence of this secondary peak demonstrates significant application potential for energy harvesting under varying wind conditions.

Wang’s [29] experiment also included a situation where the connection point was at the center of the cylinder and the inclination angle was 30°, which is similar to the conditions of our experiment. The results of this experiment show that a second peak phenomenon occurred under this condition. The starting wind speed was approximately 1.41 m/s, and the maximum voltage was between 4 V and 5 V. However, in our experiment, at the middle P3 position, no double-peak phenomenon occurred; only vortex-induced vibration was observed. The starting wind speed was 1.6 m/s, and the maximum voltage was 5.75 V. With the change in the connection point, the maximum voltage and working frequency band at P1 position were significantly improved, a double-peak phenomenon occurred at the P2 position, and the starting wind speed at P4 and P5 positions decreased. This indicates that changing the position of the connection point does indeed have an impact on the performance of the energy harvester system.

For the cause of the double-peak phenomenon at position P2, the inclination of the wind column will cause the geometric center to not coincide with the connection point. The system is simultaneously subjected to lateral force and moment excitation, triggering two modes of bending and torsion. If the frequencies of the two modes are different, the response will exhibit a bimodal characteristic: the first peak is generated by the lateral bending of the piezoelectric beam; the voltage slightly decreases during the transition stage of bending–torsion coupling; after the torsion stabilizes, the wind column can be regarded as a new blunt body; re-excitation of vortex-induced vibration occurs, and the voltage rises again. Torsion is a fluid–structure coupling effect. When the torsion frequency approaches the vortex shedding frequency, the system locks, and a new dynamic blunt body forms at the motion boundary. The equivalent Strouhal number is determined jointly by the structure and the fluid. The two voltage peaks correspond to the bending mode and the bending–torsion coupling mode respectively. After the torsion stabilizes, the new vortex shedding mode forms an independent peak in the spectrum.

**Figure 9:**
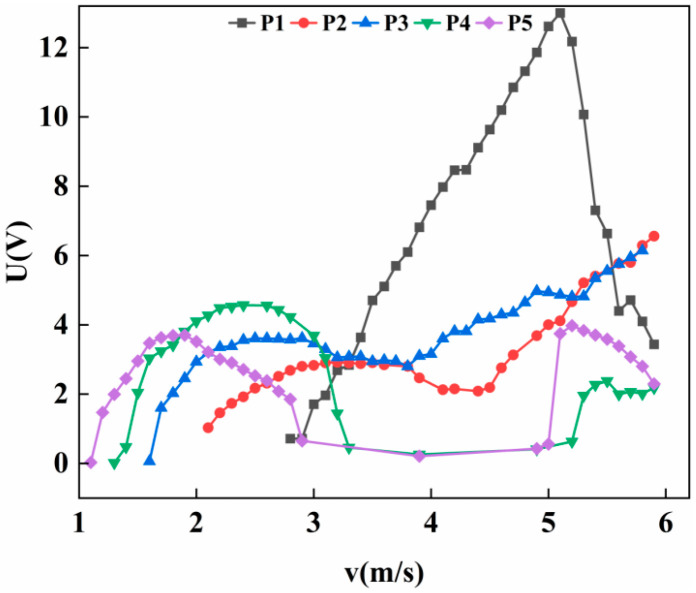
Variation of voltage with wind speed at 40° with different positions.

As shown in Figure 9, for the cylinder fixed at an inclination angle of 40°, the output voltage at position P1 increases rapidly with wind speed, reaching a peak before undergoing an equally sharp decline. At positions P2 and P3, the voltage initially increases gradually, followed by a slight decrease. As the wind speed continues to rise, a significant increase in voltage is observed, indicating the occurrence of a coupled vortex-induced vibration and galloping phenomenon. For positions P4 and P5, the voltage initially increases and then decreases to zero. As the wind speed further increases, a period of very low voltage is followed by a secondary voltage rise, resulting in a double-peak phenomenon. At position P4, the second peak shows no sign of decline by the end of the test. The maximum voltage of the first peak is 4.57 V, while that of the second peak is 2.38 V, corresponding to approximately 52.1% of the first peak. At position P5, the second peak exhibits a descending trend toward the end of the experiment. The maximum voltage of the first peak is 3.70 V, and that of the second peak is 3.97 V. The rates of voltage decline for both peaks are nearly identical, with the second peak reaching 107.3% of the first peak’s maximum voltage.

Under the experimental wind speed range of 0–6 m/s, the inclined cylinder at position P1 exhibits the highest output voltage, but also the highest cut-in wind speed. The maximum voltage occurs at 5.1 m/s; however, the voltage variation rate near this point is relatively high, indicating limited output stability. At positions P2 and P3, a coupling phenomenon between vortex-induced vibration and galloping is observed. Should the wind speed range be extended beyond 6 m/s, both the voltage output and the effective operating range at these positions are expected to improve further. At position P5, the peak voltage of the second peak exceeds that of the first, demonstrating that the double-peak phenomenon at this configuration holds significant potential for practical energy harvesting applications.

**Figure 10:**
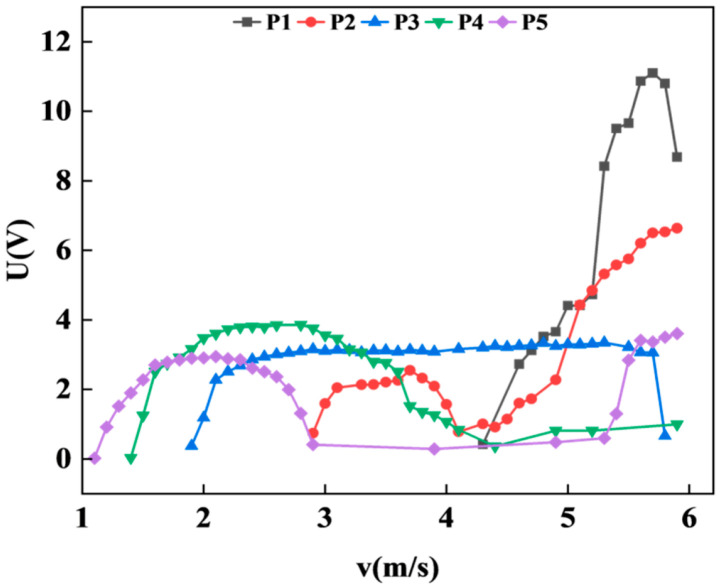
Variation of voltage with wind speed at 50° with different positions.

As shown in Figure 10, for the cylinder fixed at an inclination angle of 50°, the output voltage at position P1 exhibits a rapid increase to a maximum value, followed by an equally rapid decline, with nearly symmetric rates of ascent and descent. At position P2, the voltage initially rises, then drops sharply to near-zero levels before gradually increasing again, indicating the occurrence of coupled vortex-induced vibration and galloping. At position P3, the voltage rises rapidly, enters an extended plateau region, and finally declines sharply. The plateau phase accounts for approximately 85% of the effective wind speed range. At position P4, the voltage initially increases, experiences a brief plateau, and then slowly decreases. At position P5, the voltage first rises and then drops to zero. As the wind speed continues to increase, a period of very low voltage is followed by a secondary voltage rise, which remains ongoing by the end of the test. The maximum voltage of the first peak is 2.95 V. The voltage of the second peak reaches 3.61 V at the conclusion of the experiment, corresponding to 122.4% of the first peak’s maximum. It is anticipated that the performance at this position would be further enhanced if the wind speed range were extended.

At position P1, the inclined cylinder exhibits the highest peak voltage; however, its cut-in wind speed exceeds 4 m/s, which significantly limits its applicability under low-wind-speed conditions. As the installation position shifts from P1 to P5, the cut-in wind speed progressively decreases, reaching as low as 1.2 m/s at position P5—the lowest among all configurations. Notably, the cylinder at position P3 demonstrates a pronounced plateau phenomenon, characterized by a wide effective wind speed range and stable voltage output. This behavior makes it particularly suitable for applications where wind speed varies considerably but a consistent power supply is required. At position P5, the peak voltage of the second peak exceeds that of the first peak, indicating that the double-peak phenomenon under this configuration holds significant potential for enhancing energy harvesting performance.

Experimental observations indicate that varying the installation position significantly influences the motion state of the cylinder, thereby affecting the overall performance of the energy harvester. As illustrated in Figure 7, Figure 8, Figure 9 and Figure 10, the cut-in wind speed demonstrates a progressive decreasing trend as the installation position shifts from P1 to P5.

Although the current study did not include a direct measurement of the vortex shedding frequency, the observed voltage response can be interpreted in light of the frequency relationship and the experiment video shown in the Supplementary File. Specifically, we note that, at lower wind speeds, the vibration is dominated by the first bending mode, while at higher wind speeds, torsional modes become more prominent due to the asymmetric flow field around the inclined cylinder. This transition likely contributes to the double-peak phenomenon observed in the voltage response. The lock-in condition occurs when the vortex shedding frequency approaches the structural natural frequency. The presence of double peaks and plateau regions may correspond to transitions between bending and torsional modes, or to shifts in the lock-in range due to changes in inclination angle and installation position.

## 4. Conclusions

In this paper, an improved solution is proposed regarding the modification of the inclination angle (α) and installation position (P) of the cylindrical body relative to the incoming airflow. For this purpose, wind tunnel experiments were conducted for research. The research results indicate that different positions have a significant impact on the occurrence of the double-peak phenomenon and the plateau phenomenon. Specifically, the angles of P4 and P5 were observed to be more likely to trigger the double-peak phenomenon; the longest plateau phenomenon occurred at the P3 position of 50°. Appropriate installation positions can significantly alter the effective power generation mode of the energy harvester. The fundamental importance of the installation position lies in its ability to modulate the energy harvester’s response to wind speed: moving the attachment point from the top (P1) to the bottom (P5) systematically reduces the cut-in wind speed, while certain positions (e.g., P3 at 50°) produce an extended plateau that ensures stable voltage output over a wide wind speed range. These findings demonstrate that installation position is not merely a geometric variant but a key design parameter that can tailor the harvester’s performance for specific wind environments.

## Figures and Tables

**Table 1:** Structural and material parameters of the PEH.

Structure	Parameter	Symbol	Value	Unit
Cylinder	Length	L	13	cm
	Diameter	D	4.7	cm
	Angle	α	20/30/40/50	Degree
	Density	ρ	20	Kg·m^3^
	Weight	Mc	4.5	g
Piezoelectric layer	Length	Lp	30	mm
	Width	bp	20	mm
	Thickness	hp	0.2	mm
	Young’s modulus	Ep	62	GPa
	Permittivity	εp	35.41	nF·m^−1^
	Piezoelectric constant	ep	−17.35	C·m^−2^
	Density	ρp	7268	Kg·m^−3^
Support layer	Length	Lz	130	mm
	Width	b	30	mm
	Thickness	hs	0.5	mm
	Elastic modulus	Es	69	GPa

## Data Availability

The data that support the findings of this study are available from the corresponding author upon reasonable request.

## Supplementary Materials

The following supporting information can be downloaded at: https://www.mdpi.com/article/10.3390/mi17030372/s1. Experiment video demonstrating the operation of the energy harvester in the wind tunnel.
